# Transcriptome-wide investigation of circular RNAs in rice

**DOI:** 10.1261/rna.052282.115

**Published:** 2015-12

**Authors:** Tingting Lu, Lingling Cui, Yan Zhou, Chuanrang Zhu, Danlin Fan, Hao Gong, Qiang Zhao, Congcong Zhou, Yan Zhao, Danfeng Lu, Jianghong Luo, Yongchun Wang, Qilin Tian, Qi Feng, Tao Huang, Bin Han

**Affiliations:** National Center for Gene Research, Institute of Plant Physiology and Ecology, Shanghai Institutes of Biological Sciences, Chinese Academy of Sciences, Shanghai 200233, China

**Keywords:** *Oryza sativa*, circRNA, ssRNA-seq, ncRNA, transcriptome

## Abstract

Various stable circular RNAs (circRNAs) are newly identified to be the abundance of noncoding RNAs in Archaea, *Caenorhabditis elegans*, mice, and humans through high-throughput deep sequencing coupled with analysis of massive transcriptional data. CircRNAs play important roles in miRNA function and transcriptional controlling by acting as competing endogenous RNAs or positive regulators on their parent coding genes. However, little is known regarding circRNAs in plants. Here, we report 2354 rice circRNAs that were identified through deep sequencing and computational analysis of ssRNA-seq data. Among them, 1356 are exonic circRNAs. Some circRNAs exhibit tissue-specific expression. Rice circRNAs have a considerable number of isoforms, including alternative backsplicing and alternative splicing circularization patterns. Parental genes with multiple exons are preferentially circularized. Only 484 circRNAs have backsplices derived from known splice sites. In addition, only 92 circRNAs were found to be enriched for miniature inverted-repeat transposable elements (MITEs) in flanking sequences or to be complementary to at least 18-bp flanking intronic sequences, indicating that there are some other production mechanisms in addition to direct backsplicing in rice. Rice circRNAs have no significant enrichment for miRNA target sites. A transgenic study showed that overexpression of a circRNA construct could reduce the expression level of its parental gene in transgenic plants compared with empty-vector control plants. This suggested that circRNA and its linear form might act as a negative regulator of its parental gene. Overall, these analyses reveal the prevalence of circRNAs in rice and provide new biological insights into rice circRNAs.

## INTRODUCTION

In the essential thesis of molecular genetics, mature messenger RNAs (mRNAs) are linear molecules with clear termini. In past years, circular RNAs (circRNAs), which are formed by 3′–5′ ligation in a splicing reaction of a single RNA molecule, have been only occasionally identified due to the limitation of molecular techniques and bioinformatic tools (Sanger et al. 1976; Hsu and Coca-Prados 1979; Grabowski et al. 1981; Kos et al. 1986; Nigro et al. 1991). Despite their existence in both unicellular and multicellular organisms, circRNAs were previously considered rare in nature or even disregarded as transcriptional noise or artifacts.

Recently, knowledge of circRNAs has changed substantially. With the advent of high-throughput sequencing technology and high-efficiency big data analysis, an abundance of circRNAs has been identified in Archaea, *Caenorhabditis elegans*, mice, and humans (Danan et al. 2012; Salzman et al. 2012; Memczak et al. 2013; Zhang et al. 2013, 2014). In Archaea, by the construction of RNase R-treated cDNA libraries and RNA-seq (termed circRNA-seq), 37 circRNAs were detected (Danan et al. 2012). In humans, by RNA-seq from a variety of normal and malignant cells, strong evidence demonstrates that thousands of genes can be expressed in the form of circRNAs (Salzman et al. 2012). In human fibroblasts, more than 25,000 distinct RNAs have been validated as exonic circular RNAs (ecircRNAs) and determined to be more stable than the associated linear mRNAs in vivo (Jeck et al. 2013). Recently, 1950, 1903, and 724 circRNAs have been detected in human, mice, and *C. elegans*, respectively (Memczak et al. 2013). Another 103 RNase R-enriched intronic circular RNAs have also been identified in human H9 cells (Zhang et al. 2013).

Though it is considered that circRNAs lack poly(A) tails, some circRNAs have been detected in libraries prepared from poly(A)-selected RNAs (Lamm et al. 2011; Salzman et al. 2012). CircRNAs often show tissue- or developmental stage-specific expression. Furthermore, the evidence suggests that circRNAs play important biological roles in transcriptional and post-transcriptional regulation (Chen and Sarnow 1995; Chao et al. 1998; Gualandi et al. 2003; Ebert et al. 2007; Franco-Zorrilla et al. 2007). CircRNA is also reported as a class of competing endogenous RNA moleclules (ceRNAs) that stably sequester microRNAs (miRNAs) to terminate suppression of their mRNA targets (Hansen et al. 2011, 2013; Memczak et al. 2013). However, other research provides strong evidence demonstrating that no other circRNA stands out as a candidate to act as a strong sponge for any of the other RNA-binding proteins (Guo et al. 2014).

CircRNA production mechanisms have also been investigated. Zhang et al. (2014) demonstrate that exon circularization is dependent on flanking intronic complementary sequences. Moreover, the efficiency of exon circularization is regulated by competition between RNA pairing across flanking introns or within an individual intron (Zhang et al. 2014).

In contrast to recent mammalian research, to date, circRNAs in plants have not been comprehensively identified, although some circRNA research has been carried out for *Arabidopsis* (Wang et al. 2014). Rice (*Oryza sativa* L.) is among the most important crops and an excellent monocotyledonous model plant. To understand the exact quantity of circRNAs and their potential function in the regulation of rice gene expression, we have systematically investigated circRNAs in rice using high-throughput strand-specific RNA sequencing technology (ssRNA-seq), third-generation sequencing technology (TGS), experimental approaches, and bioinformatic tools. Here, we deep-sequenced cDNAs and comprehensively screened ssRNA-seq data to identify circRNAs in *O. sativa* ssp. *japonica* Nipponbare. All mRNAs were derived from mature leaves and panicles grown under normal conditions and were individually extracted in poly(A)-selected and poly(A)-depleted samples. We further developed a computational pipeline to identify backsplice sites by mapping paired-end reads to the genomic sequence. We reported hundreds of circRNAs from both poly(A)-selected and poly(A)-depleted samples in rice. We validated their stable expression and circularization using the predicted backsplice sites. Certain circRNAs appeared to be specifically expressed across different tissues. The results suggested that multiple circRNA isoforms produced from a single parental gene (alternative circularization) might be prevalent in rice. In contrast to exonic circRNAs in humans (Hansen et al. 2013; Memczak et al. 2013), our research shows that circRNAs in rice have little enrichment for miRNA target sites. Moreover, overexpression constructs in rice suggested that circRNAs might act as negative regulators of their parental genes. Together, our data provide the first genome-wide profiling of circRNAs in rice and reveal their widespread occurrence and potential important biological roles in transcriptional and post-transcriptional regulation.

## RESULTS

### Identification of circRNAs in rice

**FIGURE 1.**
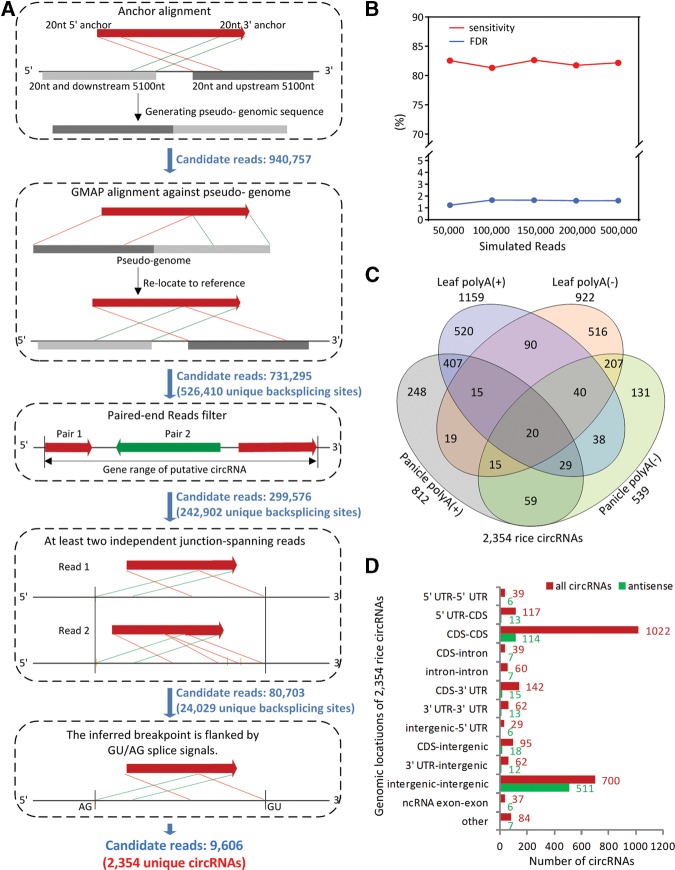
Identification and classification of rice circRNAs. (*A*) Flowchart for identification of circRNAs. (GMAP) A genomic mapping and alignment program. (*B*) Sensitivity and false-discovery rate estimates. Referring to RAP2 gene models, reads with backsplice sites were simulated. A complete analysis of five individual data sets (∼1500–10,000 designed backsplice sites) yielded a sensitivity of >81.4% and a FDR of <1.7%. (*C*) Venn diagrams of circRNAs detected in poly(A)-selected and poly(A)-depleted samples of rice leaf and panicle. (*D*) Genomic origin of rice circRNAs. The numbers in red represent genomic annotation of all 2354 circRNAs referring to RAP2. The numbers in green represent some of all circRNAs that could align in an antisense manner to other genes.

To obtain sufficient transcriptome data, we separately deep-sequenced poly(A)-selected and poly(A)-depleted samples with technical and biological duplicates from the mature leaf and panicle tissues of *O. sativa* ssp. *japonica* Nipponbare. These samples were sequenced using the Illumina ssRNA-seq approach, yielding a total of 710 million paired-end reads sized 100 bp with orientation accuracy from 92.7% to 98.3% that mapped uniquely to the rice reference genome (IRGSP v5.0) (Supplemental Table 1). To comprehensively investigate stably expressed circRNAs in rice, we focused on identifying a key feature of circRNAs, an out-of-order arrangement of exons referred to as backsplicing (Materials and Methods). First, we extracted reads that were uniquely but partially mapped to the genome (20.1% of mapped reads, 285,823,960 single reads). From this preliminary screening reservoir, we then collected those reads in which both terminal regions (20 bp) could be perfectly and uniquely anchored to the same chromosome sequence in a permuted, chiastic order. In this step, we obtained 940,757 candidate reads (Fig. 1A). Second, for each candidate, according to its anchored positions, downstream and upstream sequences were reverse-assembled into a pseudo-genome. In this step, we could search for backsplices in the traditional manner of aligning cDNA sequences to genomes. We were able to detect 731,295 reads corresponding to 526,410 unique backsplices, suggesting that circular products were prevalent in ssRNA-seq samples. Furthermore, referring to the genomic positions of their paired-end reads, we filtered out sequences mapped outside of the backspliced exons which could not be explained by a circRNA (299,576 reads corresponding to 242,902 unique backsplices were retained). Finally, we required that each backsplice be supported by at least two independent junction-spanning reads and that the backsplice junction be flanked by the GU/AG intron signal. In total, based on this computational pipeline, we identified 2354 unique circRNAs from poly(A)^+^ and poly(A)^−^ ssRNA-seq data in rice (Supplemental Table 2). We estimated the false-discovery rate (FDR < 1.7%) and sensitivity (>81%) using five individual simulated data sets (Materials and Methods and Fig. 1B). We also detected candidate reads using another mapping program “Segemehl” (Hoffmann et al. 2014), showing that 92.2% of candidate reads overlapped with the ones we identified.

To investigate whether circRNAs exist in other plants, we downloaded public RNA-seq data and detected 75, 113, 496, 26, and 165 circRNAs in the monocot plants *Sorghum bicolor*, *Setaria italica*, *Zea mays*, and *Brachypodium distachyon* and the dicot model plant *Arabidopsis thaliana*, respectively (Supplemental Tables 3, 4). Obviously, the depth of publicly available transcriptome sequences of other plants was much less than that of rice. The sequencing coverage of rice transcriptome was between 129 and 310, while in other monocot plants, the sequencing coverage was only between 3 and 15 (Supplemental Table 4). This indicated that identification of circRNAs required more sequencing depth, preferably hundreds of millions of reads, even in libraries with preferential enrichment by RNase R digestion. In addition, of these circRNAs, 50 could be mapped to rice circRNAs with >100 bp and >80% sequence identity.

### Properties of rice circRNAs

To evaluate the prevalence of circRNAs, we analyzed the biological replicates of panicles and leaves. Although gene expression levels were largely consistent (Supplemental Fig. 1A), the number of overlapping circRNAs in each biological replicate was relatively small (Supplemental Fig. 1B). This result indicated that reads with head-to-tail junctions were randomly sequenced. In total, only 20 circRNAs were identified in all four samples and 939 (39.9%) were identified in at least two of the four samples (Fig. 1C). The total numbers of detected circRNAs in poly(A)-selected samples [1159 in leaf poly(A)^+^ and 812 in panicle poly(A)^+^] are slightly higher than those in poly(A)-depleted samples [922 in leaf poly(A)^−^ and 539 in panicle poly(A)^−^]. This might be because more high quality transcriptome data were obtained in poly(A)-selected samples than in poly(A)-depleted samples (Supplemental Table 1). However, the detection efficiency of circRNAs in poly(A)-depleted samples (153–181 candidates for one circRNA) is much higher than that in poly(A)-selected samples (314–415 candidates for one circRNA) (Supplemental Fig. 1C).

We annotated rice circRNAs with the reference of RAP2 gene models. Information on circularized exons and their flanking introns was extracted from existing gene annotations. The majority of backsplices (81.7%) spanned one to four exons. In contrast, 39.0% of their parental genes had more than ten exons (Supplemental Fig. 1D). The mean length of exonic circRNAs was 474 bp, which is much shorter than the length of their parental (resident) genes (2188 bp) (Supplemental Fig. 1E). The dominant circularizing events comprised coding sequence (CDS–CDS)-originating (1022) and intergenic-originating (700) circRNAs (Fig. 1D). In total, 1356 circRNAs (55.5%) were derived from exons (exonic circRNA); that is, both backsplice sites coincided with known exonic boundaries or fell into an exon of the best matching transcript, which included CDS–CDS (1022), 5′ UTR–5′ UTR (39), 5′ UTR–CDS (117), CDS-3′ UTR (142), and 3′ UTR-3′ UTR (62) circRNAs. Another 700, 95, 62, 60, and 37 circRNAs were derived from intergenic regions, CDS-intergenic, 3′ UTR-intergenic, introns, and ncRNAs, respectively. Other smaller fractions aligned across two genes or two different genomic components, which included 39 exon–intron circRNAs. The results showed that 927 circRNAs use at least one previously annotated splice site. Of 927 circRNAs, 484 of these had two known splice sites involved in circularization. That is, 60.6% (1427) of rice circRNAs use two novel backsplices (Supplemental Table 2). Excluding those from intergenic and intronic regions, it was notable that 325 circRNAs had only one previously annotated splice site; and 539 circRNAs with both novel backsplices were exonic ones. Additionally, 730 circRNAs, including 511 intergenic–intergenic circRNAs and 114 CDS–CDS circRNAs, could also align in an antisense manner to known transcripts (Fig. 1D).

Because a considerable amount of circRNAs were partially or fully located in intergenic regions according to RAP2 gene models, it was necessary to further investigate whether these novel circRNAs had their parental linear mRNAs or not. For this purpose, we used the TGS (PacBio *RS* II) sequencing platform to capture the full-length mRNA in leaf because PacBio can generate long reads with an average read length of 3 kb (English et al. 2012). We generated a full-length cDNA library by an established method using the Clontech SMARTer PCR kit. The prepared cDNA samples were then converted to libraries for PacBio single-molecule real-time sequencing. Overall, 2,740,632 raw reads were generated. A total of 145,505 error-corrected long reads were obtained by SMRT Analysis v2.3 Software. After removing adaptor sequences and filtering shorter than 50-bp reads, 143,010 reads with N50 of 860 bp, mean length of 738 bp, and maximum length of 6457 bp were collected. Of them, 141,927 (99.2%) reads could be properly aligned to the reference rice genome (IRGSPv5.0) by GMAP; 125,987 (88.1%) reads could be mapped to 14,555 RAP2 gene models. Therefore, 15,940 long reads were defined as novel transcripts. Compared with 998 novel circRNAs by excluding 1356 exonic circRNAs, 92 circRNAs were fully located in 76 novel transcripts with the same transcriptional orientations, indicating that at least 9.2% of them had parental linear mRNAs.

### Validation of rice circRNAs

**FIGURE 2.**
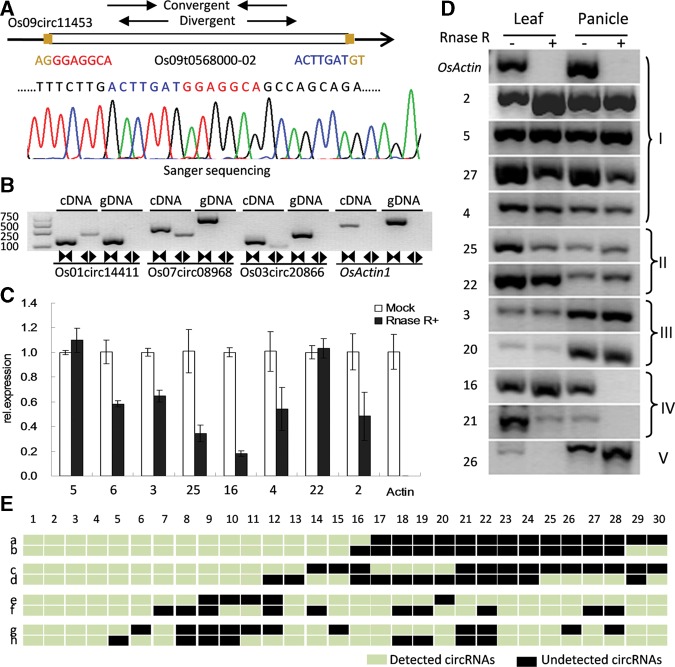
Various experimental strategies validated the stable expression of rice circRNAs. (*A*) An example of rice circRNAs (Os09circ11453) shows the validation strategy. Divergent and convergent primers were designed to detect circular RNAs. Sanger sequencing further confirmed head-to-tail backsplicing. (*B*) Divergent primers (black back-to-back triangle pairs) successfully amplified three circRNAs (Os01circ14411, Os07circ08968, and Os03circ20866) in cDNA but failed to do so in genomic DNA. Convergent primers (black opposing triangle pairs) worked on both cDNA and genomic DNA. (OsActin1) Linear control. (*C*) Here, qRT-PCR shows that eight circRNAs are stably expressed in both RNase R(−) and RNase R(+) total RNA samples, whereas the linear control was not expressed in RNase R(+) samples. (*D*) RT-PCR further shows that 11 circRNAs are RNase R-resistant. Some circRNAs showed tissue-specific expression. (I) circRNAs highly expressed in both tissues; (II) circRNAs highly expressed only in leaf; (III) circRNAs highly expressed only in panicle; (IV) circRNAs specifically expressed in leaf; and (V) circRNAs specifically expressed in panicle. (*E*) Expression results of 30 circRNAs in leaf and panicle poly(A)^−^ and poly(A)^+^ samples, summarized according to ssRNA-seq data and RT-PCR. (a) Results obtained from leaf poly(A)^+^ ssRNA-seq; (b) results obtained from leaf poly(A)^+^ RT-PCR; (c) results obtained from panicle poly(A)^+^ ssRNA-seq; (d) results obtained from panicle poly(A)^+^ RT-PCR; (e) results obtained from leaf poly(A)^−^ ssRNA-seq; (f) results obtained from leaf poly(A)^−^ RT-PCR; (g) results obtained from panicle poly(A)^−^ ssRNA-seq; and (h) results obtained from panicle poly(A)^−^ RT-PCR. Note: The name of each circRNA has been indexed in Supplemental Table 5 and Supplemental Figure 2.

As described above, we identified 2354 novel circRNAs in poly(A)-selected and poly(A)-depleted samples from the mature leaf and panicle tissues. Of them, a few exonic circRNAs use one novel backsplice (325 circRNAs) or two novel backsplices (539 circRNAs). We further experimentally tested the predictions of rice circRNA including its expression and back splicing sites. Divergent primers and convergent primers were designed to amplify each circRNA in the total RNA sample, RNase R-treated sample (containing degraded linear RNA molecules), and genomic DNA. Theoretically, unlike convergent primers, divergent primers could amplify the circular form and also be resistant to RNase R, which could rule out the other noisy backsplices produced by trans-splicing, genomic rearrangements, or potential PCR artifacts. The predicted backsplices of 30 out of 35 randomly chosen circRNAs (85.7%) were successfully validated (Supplemental Fig. 2), including 24 exonic, four intergenic, one intronic, and one cross-genic circRNAs. In addition, 15 of these circRNAs had two known backsplices, 10 had two novel backsplices, and five had one novel backsplice. Head-to-tail junctions were amplified only in cDNA samples and further detected by Sanger sequencing (Fig. 2A,B; Supplemental Fig. 2). Likewise, novel circRNAs located in intergenic or intronic regions were also validated by sampling (Fig. 2A; Supplemental Fig. 2). We quantified RNase R resistance for eight confirmed circRNAs by qRT-PCR. All circRNAs were at least 20-fold more resistant than the control marker (Fig. 2C). Of 30 experimentally validated circRNAs, 16 could be detected in both leaf and panicle with nearly equal expression, five were more highly expressed in leaf, two were more highly expressed in panicle, three were panicle-specific, and another four were leaf-specific (Supplemental Fig. 2). The results obtained from semiquantitative PCR further confirmed the tissue-specific expression of certain circRNAs (Fig. 2D). In addition, using these 30 circRNAs, we experimentally tested our predicted circRNAs in poly(A)-selected samples and poly(A)-depleted samples. The results showed that 27, 17, 19, and 21 out of 30 candidates from leaf poly(A)^+^, panicle poly(A)^+^, leaf poly(A)^−^, and panicle poly(A)^−^, respectively, were consistent with our predictions (Fig. 2E).

In addition, we carried out Northern blot analysis of total RNAs from panicle and leaf with or without RNase R-treatment to confirm the presence of antisense genes of circRNA “Os02circ00436” (Supplemental Fig. 3). The results showed that Os02circ00436 was enriched in RNase R-treated samples, and it could only be detected by sense probe in the hybridization.

### Alternative circularization of rice exonic circRNAs

**FIGURE 3.**
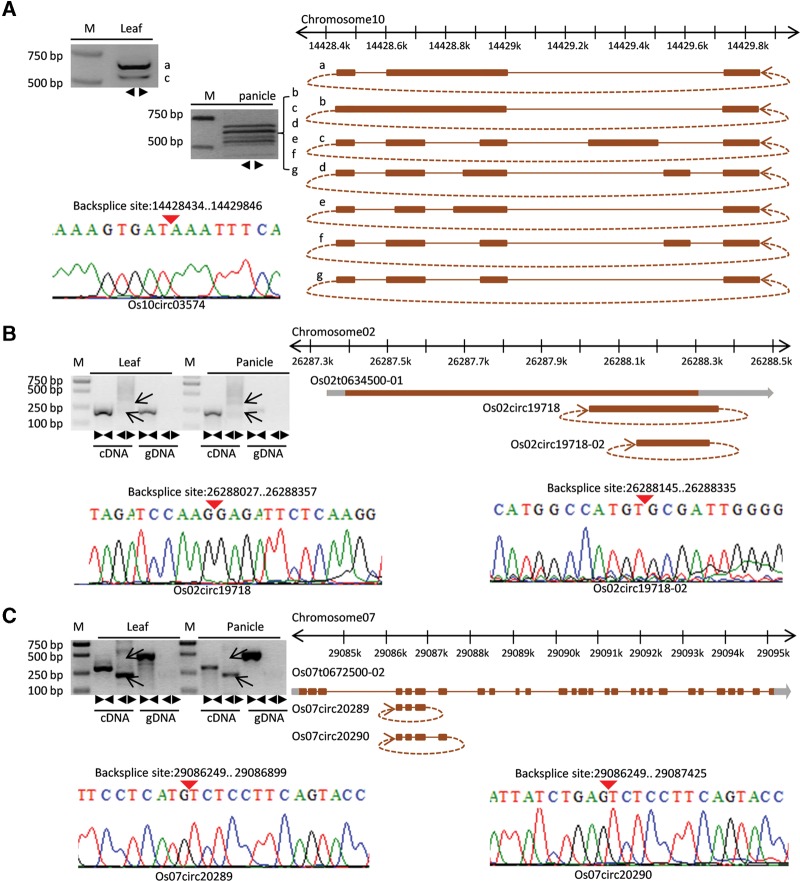
Visualization and validation of three rice alternative circularization events. (*A*) In the *upper left*, the PCR amplifications with divergent primers of circRNA “Os10circ03574” in leaf (two bands are indicated) and panicle (six bands are indicated) were performed. In the *lower left*, the backsplice site was further confirmed by Sanger sequencing. The sequence was visualized using a raw trace file. Red *inverted* triangles indicate the junction loci. On the *right*, seven alternative splicing circularization structures of “Os10circ03574” obtained from Sanger sequencing are shown in detail. A brown bar denotes an exon, a gray bar denotes UTR, and a brown line denotes an intron. (*B*,*C*) Similar to *A*, the results from PCR amplification and Sanger sequencing are displayed. Alternative backsplicing circularization events were identified and validated.

Circularization or the lack of circularization was determined only by backsplicing sites, and therefore we inferred the gene structure of circRNAs based on annotated transcripts. To avoid the occurrence of fuzzy gene structure, only exonic circRNAs were included in such analyses. The corresponding exon boundaries of the circRNA were trimmed on the basis of backsplice sites. Among 1356 exonic circRNAs, we identified 486 alternative backsplicing circularization events originating from 175 unique parental gene loci. In total, 115 of 175 genes produced two different circRNA isoforms, 32 produced three distinct isoforms, 13 produced four isoforms, and another 15 produced at least five distinct ecircRNAs. In addition to alternative backsplicing circularization patterns (same parental gene, different backsplices), we also experimentally identified alternative splicing circularization events (same backsplice, different transcription structure) (Fig. 3). The circRNA “Os10circ03574” was located in an intergenic region based on RAP-DB annotation. PCR amplification produced from divergent primers followed by Sanger sequencing validated the predicted backsplice site and further identified a total of seven isoforms in leaf and panicle (Fig. 3A). Interestingly, only two isoforms (type a and type c) were found in leaf, while six isoforms (types b to g) could be found in panicle. This result indicated that some alternative circularization events were tissue specific. In addition, a circRNA with a novel backsplice site (Os02circRNA19718-02) from the same parental gene was identified (Fig. 3B,C displays an example of two alternative splicing circularization events originating from known junctions).

In mammals, “direct backsplicing” (also termed intron-pairing-driven circularization) has been proposed to be a common mechanism for ecircRNA formation (Salzman et al. 2012; Jeck et al. 2013; Jeck and Sharpless 2014). Recently, in humans, it has been demonstrated that exon circularization depends on flanking intronic complementary sequences. Further evidence showed that competition between inverted repeated Alu pairs can lead to alternative circularization (Zhang et al. 2014). To determine whether a similar production mechanism occurs in rice, we detected putative intronic complementary sequences by testing 500-bp flanking sequences of the backsplice sites from 2354 rice circRNAs. Only 20 circRNAs were selected with at least 18-bp flanking intronic complementary sequences. Furthermore, we downloaded plant miniature inverted-repeat transposable elements (MITEs) from the P-MITE database (Chen et al. 2014) to compare their genomic locations with those of the flanking sequences of circRNAs in rice. In total, only 72 of 2354 circRNAs were determined to have two flanking sequences that overlapped with MITEs. Therefore, other common mechanisms in rice may be involved in ecircRNA generation in addition to intron-pairing-driven circularization.

### Putative functions of rice circRNAs

To detect whether rice circRNAs could affect gene post-transcriptional regulation by binding to miRNAs and preventing them from regulating their target mRNAs, which was demonstrated for some human circRNAs (Memczak et al. 2013), we identified circRNA-originating target mimics in rice. We found that 235 of 1356 ecircRNAs had putative miRNA-binding sites. Of these 235, only 31 circRNAs had two to six miRNA-binding sites, which was significantly less than reported for the human circRNA CDR1as.

**FIGURE 4.**
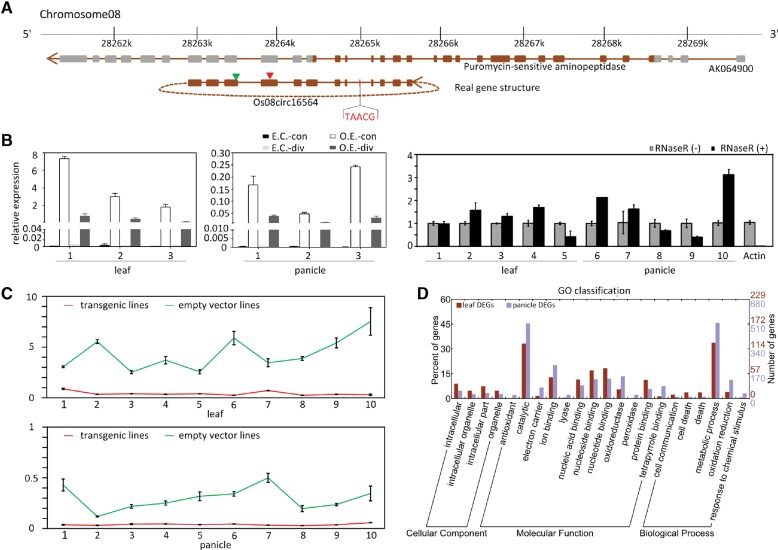
Analysis of the overexpression of the circRNA “Os08circ16564” in transgenic rice plants. (*A*) Visualization of Os08circ16564 circularization structure and its parental gene (AK064900). A brown bar denotes an exon, a gray bar denotes UTR, and a brown line denotes an intron. Vertical triangles indicate the predicted miRNA osa-miR810b.2 (red) and osa-miR172d-5p (green) binding sites. (*B*) The qRT-PCR results of Os08circ16564 overexpression lines and empty-vector transgenic lines. Leaf (*left* panel) and panicle (*middle* panel) tissues were collected from three independent lines. (E.C.-con) Convergent PCR for empty-vector control transgenic plants; (O.E.-con) convergent PCR for overexpression transgenic plants; (E.C.-div) divergent PCR for empty-vector control transgenic plants; (O.E.-div) divergent PCR for overexpression transgenic plants. (*Right* panel) The qRT-PCR results of Os08circ16564 by divergent primers in overexpression transgenic plants. *Actin* was used as a linear control. Leaf and panicle tissues were collected from five independent rice plants. Error bars indicate ± SD. (*C*) The qRT-PCR results show that the expression levels of Os08circ16564's parental gene AK064900 in both the leaf and panicle tissues of the Os08circ16564-transgenic plants were greatly reduced compared with those of empty-vector transgenic plants. Leaf and panicle tissues were collected from 10 lines. Error bars indicate ± SD. (*D*) Functional classification of DEGs between transgenic and empty-vector transgenic lines in leaf and panicle.

Although some putative miRNA-binding sites in circRNAs have been identified, experimental evidence for the functional analysis in rice is still lacking. Since miR172 plays a crucial role in the development of the rice spikelet and floral organ (Zhu et al. 2009), we performed a transgenic analysis for an overexpression of a circRNA Os08circ16564, which was predicted to be a target mimic of miR172 and miR810 (Fig. 4A).

To generate the circRNA overexpression construct, we first cloned the Os08circ16564 exons from leaf cDNA samples of *japonica* Nipponbare into the multiple cloning sites of the binary vector pTCK303 under the driving of a maize *ubiquitin* promoter (Wang et al. 2004). Second, we inserted an intron DNA fragment of a rice gene, which was not expressed, into both upstream and downstream flanking Os08circ16564 exons in an orientation-opposite pattern (Supplemental Fig. 5A). According to the report by Zhang et al. (2014), the nonrepetitive complementary sequence can promote circRNA formation. Therefore, the Os08circ16564 exons would form a loop under the help of a hairpin structure that was generated from the inverted complementary sequence (Supplemental Fig. 5C[ii]). Furthermore, the hairpin was cleaved by Dicer into small RNA duplexes and a terminal loop. The application of such a construct allowed us to visualize the overexpression of Os08circ16564 by quantitative RT-PCR.

To detect whether the overexpression construct could generate linear forms or circular forms of Os08circ16564 or both, we first used a pair of convergent primers to detect the total transcripts of Os08circ16564. The convergent primers were anchored on one side of the vector and the other side on the exons of Os08circ16564; therefore these primers allowed only detecting transcripts from the transgenic Os08circ16564 construct (Supplemental Fig. 5A). The results showed that the exogenous Os08circ16564 was not detected by convergent primers in empty-vector control plants, whereas a large amount of transcripts were detected in Os08circ16564-transgenic plants (Fig. 4B). Similar expression patterns were detected in the leaf and panicle samples (Fig. 4B). Next, we used a pair of divergent primers to detect the circular forms of Os08circ16564. In empty-vector control plants, the endogenous circular Os08circ16564 were barely detected. In contrast, the circular forms were found to be more abundant in Os08circ16564-transgenic plants, and showed RNase R-resistance (Fig. 4B). It should be noted that the increased circular forms were taken from ∼8%–23% of the total transcripts compared with the relative expression level of the PCR product generated by divergent primers and convergent primers. This result indicated that the transgenic circularization efficiency was not as high as expected. This phenomenon might also be due to the degradation of circRNAs into linear forms.

In addition, when validating the transgenic product by divergent PCR on an agarose gel, we found that there were three overexpressed bands compared to the control plant (Supplemental Fig. 5B,C[i]). Sanger sequencing showed that the three transcripts were all circular forms. However, all of them missed some sequences compared with the Os08circ16564 (Supplemental Fig. 5C[iii]). We suspected that the Dicer enzyme did not cut the stem–loop like RNAs at the site as we expected. However, the micro172-binding sites were retained in all of them.

We also investigated miR172 expression in overexpressed Os08circ16564-transgenic plants. The quantitative RT-PCR results showed that there was no significant difference of miR172 expression levels between empty-vector control plants and Os08circ16564-transgenic plants (Supplemental Fig. 4), indicating that circRNA might not be the sponge of miRNAs in rice.

In view of another report showing that the exon–intron circRNAs could promote the expression of their parental genes (Li et al. 2015), we next detected the parental genes expression on the transgenic plants. We examined the expression level of the Os08circ16564 parental gene AK064900 in both leaf and panicle tissues in 10 independent Os08circ16564-transgenic and empty-vector control transgenic rice plants. Surprisingly, in the Os08circ16564-transgenic rice plants, the expression level of AK064900 was greatly reduced in both the leaf and the panicle compared to the control plants (Fig. 4C). Considering there existed a large amount of linear form transcripts detected in the overexpressed transgenic plants, we would suggest that circRNAs and its linear forms might act as post-transcriptional regulators of their parental genes.

To further discover the mechanism of the down-regulation of AK064900 caused by overexpression of Os08circ16564, we next screened out the differentially expressed genes (DEGs) between transgenic and nontransgenic lines and applied ssRNA-seq to sequence the rice cDNAs of four samples (transgenic leaf, nontransgenic leaf, transgenic panicle, and nontransgenic panicle). In total, we identified 636 and 1499 DEGs in the leaf and panicle (Supplemental Table 6). Of these DEGs, the expression levels of 234 leaf and 977 panicle genes were down-regulated in transgenic lines, whereas 402 leaf and 522 panicle genes were up-regulated in transgenic lines. Only 55 DEGs were identified in both leaf and panicle tissue. GO classification of the DEGs showed that these genes are involved in a broad range of molecular functions, such as catalytic, ion binding, nucleotide binding, nucleoside binding, and oxidoreductase functions (Fig. 4D). The major biological processes in which DEGs were involved were metabolic processes.

## DISCUSSION

In the last few years, functional annotations of the rice transcriptome have been used to make great progress in documenting the whole-genome transcriptome profiles (Lu et al. 2010). Although thousands of distinct circRNAs have been discovered in various cell types in mammals and many of these circRNAs have been found to be abundant and stable, little is known regarding plant circRNAs. We report here the first genome-wide identification and potential function analysis of circRNAs in plants.

Our study significantly augments the level of knowledge of circRNAs in plants. We provide strong evidence that rice circRNAs have a large number of isoforms, including alternative backsplicing and alternative splicing circularization patterns. We found that alternative circularization was prevalent in rice and that parental genes with multiple exons are preferentially circularized. We also found that some circRNAs were expressed specifically across different tissues. We believe that the actual number of circRNAs in rice might be underestimated because we analyzed the sequencing data of only two normal tissues (leaf and panicle) with a stringent searching approach. Collecting more tissues from various developmental periods grown under normal and alternative treatment conditions is necessary to identify more circRNAs. Furthermore, circRNA analysis requires sufficient sequencing depth, preferably hundreds of millions of reads, even in libraries with preferential enrichment by RNase R-digestion. As shown in Supplemental Table 4, the numbers of circRNAs of other plants that were identified from low-sequencing coverage data were much lower than those of rice.

We experimentally validated the novel backsplice sites by divergent primer amplification and Sanger sequencing. Likewise, novel circRNAs located in intergenic or intronic regions were also validated by sampling. In addition, apart from the 1356 ecircRNAs, the precise gene structures of the remaining 998 circRNAs were restricted to current genome annotation data. Therefore, we are not sure if these novel backsplice sites are specific to circRNAs or are also found in their parental linear mRNAs. Our early study (Lu et al. 2010) showed that quite a few novel transcriptional active regions could be identified using RNA-seq. However, because of short sequencing reads (75 bp), precise gene structures could not be assembled. To evaluate this problem, we further used PacBio *RS* II, the TGS technology sequencing platform that can generate long reads, to capture the full-length mRNA in rice. Of 143,010 error-corrected reads, 15,940 long reads could be defined as putative novel transcripts. Compared with 998 novel circRNAs by excluding 1356 ecircRNAs, 92 circRNAs were fully located in 76 novel transcripts with the same transcriptional orientations, which indicated that at least a few circRNAs had parental linear mRNAs.

New research has found that only a few circRNAs exhibit more miRNA sites than would be expected by chance (Guo et al. 2014). Our results also showed no significant enrichment for miRNA target sites in rice circRNAs. To determine the circRNA production mechanism in rice, we detected putative intronic complementary sequences from 2354 rice circRNAs. Our data showed that only a very small proportion of rice circRNAs have two flanking sequences overlapping with MITEs. Thus, we concluded that other common mechanisms in rice might be involved in ecircRNA generation in addition to intron-pairing-driven circularization. This is to be further investigated.

Some circRNAs have been shown to act as miRNA sponges and positive regulators of their parental coding genes (Danan et al. 2012; Salzman et al. 2012; Memczak et al. 2013; Zhang et al. 2013, 2014). In this analysis, we conducted a transgenic study through the overexpression of a circRNA construct to investigate its biological function. Os08circ16564 was predicted as a target mimic of miR172, and an overexpression construct was therefore generated for transgenic analysis. It was hypothesized that miR172 plays a crucial role in the development of the rice spikelet and floral organ (Zhu et al. 2009). Although no clear change in the phenotype, including in the spikelet or floral organ, could be detected in the transgenic plants, we found that the expression level of the parental gene AK064900 was highly reduced both in leaf and panicle compared to empty-vector control transgenic plants. Considering that there existed a large amount of linear form transcripts detected in the overexpressed transgenic plants, we would suggest that circRNAs and its linear forms might act as negative post-transcriptional regulators of their parental genes, although the biological mechanisms are still unknown. Since there is no significant difference in the expression levels of miR172 between transgenic and control lines, we have no evidence to show that rice circRNAs act as miRNA sponges. Collectively, our results indicate that circRNAs are prevalent and stably expressed in rice. Our analysis provides new biological insights into rice circRNAs.

## MATERIALS AND METHODS

### Plant materials

Seeds of cultivated rice *Oryza sativa* ssp. *japonica* Nipponbare were germinated at 30°C in an incubator, and all plants were grown in the paddy field of the Shanghai Institute of Plant Physiology and Ecology, Shanghai, China. Panicles and mature leaves were harvested at the flowering time for RNA isolation.

### Genomic DNA, total RNA extraction, and RNase R treatment

Genomic DNA was extracted from fresh tissue using the cetyl-trimethylammonium bromide method (Murray and Thompson 1980) with minor modifications. Genomic DNA was used as a negative control for divergent primers.

Total RNA was isolated from all samples using TRIzol reagent (Invitrogen) according to the manufacturer's protocol. To remove polysaccharide contamination, 0.25 mL of isopropanol was added to the aqueous phase, followed by 0.25 mL of a high-salt precipitation solution (0.8 M sodium citrate and 1.2 M NaCl) per 1 mL of TRIzol reagent to precipitate RNA. Total RNA samples were treated with DNase I (NEB) and purified by RNA Clean & Concentrator-25 spin columns (ZYMO Research) to remove DNA contamination and salts. RNA was evaluated using 2.0% TAE–agarose gel electrophoresis.

For RNase R-treated total RNA samples, the purified DNase I-treated total RNA was incubated for 15 min at 37°C with 3 units µg^−1^ of RNase R (Epicentre). RNA was subsequently purified by phenol–chloroform extraction and re-precipitated in three volumes of ethanol.

### PCR amplification and Sanger sequencing

The PCR primers (divergent and convergent) were designed for circRNA validation (Supplemental Table 5). The cDNA retro-transcribed from total RNA treated with DNase I and RNase R and the genomic DNA were used as templates. Convergent primers were used as positive controls for linear transcripts, and divergent primers were used to detect the candidate circular template. For each PCR amplification, 20 ng of cDNA or genomic DNA was used with rTaq DNA polymerase and 10× buffer (Takara). We performed 35 cycles of PCR. To confirm the PCR results, the PCR products of the expected length were dissected from a gel and purified using the QIAquick Gel Extraction Kit (Qiagen). Direct sequencing was performed on an ABI 3730 sequencer according to the manufacturer's protocol.

### Semiquantitative and quantitative real-time PCR

RNase R-treated total RNA samples and untreated RNA samples were retro-transcribed with random primers using SSIII reverse transcriptase (Invitrogen). For semiquantitative PCR, divergent PCR products were amplified for 36 cycles, while rice OsActin1 (AK100267) products were amplified for 26 cycles. For qRT-PCR, diluted first-strand cDNA was amplified using the SYBR Green PCR MasterMix (Takara) according to the manufacturer's instructions. qRT-PCR was performed on an Applied Biosystems 7500 real-time PCR System. The levels of circRNA transcripts were normalized to endogenous linear OsActin1 transcripts. Each set of experiments was repeated three times. The primers used for semiquantitative and quantitative RT-PCR are listed in Supplemental Table 5.

### ssRNA-seq library preparation and Illumina sequencing

A modified protocol from our previous work was used to produce sequencing libraries (Lu et al. 2012). Total RNA was depleted of ribosomal RNA using the RiboMinus Plant Kit (Invitrogen). Then the Dynabeads mRNA Purification Kit (Ambion) was used to purify poly(A)^+^ mRNA from the rRNA-depleted RNA samples. This protocol relies on base-pairing between the poly(A) residues at the 3′ end of most mRNA and the oligo (dT)_25_ residues covalently coupled to the surface of the Dynabeads. RNA lacking a poly(A) tail will not hybridize to the beads and are readily washed away. The remainder was thus further prepared as poly(A)^−^ samples. RNA was then fragmented using an RNA fragmentation kit (Ambion). We prepared a cDNA library by following an established protocol (Parkhomchuk et al. 2009). Next, the sequencing libraries were constructed according to the manufacturer's instructions (Illumina). Size selection of 300–400 bp was performed, and the resulting samples were purified and then enriched by PCR for 15 cycles. Samples of leaf poly(A)^+^, leaf poly(A)^−^, panicle poly(A)^+^, and panicle poly(A)^−^ were loaded into 5, 5, 5, and 4 flow cell channels of a HiSeq 2000 Sequencer, respectively, at a concentration of 2 pM for 2 × 100-bp paired-end sequencing.

### Full-length cDNA library preparation and PacBio *RS* II sequencing

SMARTer PCR cDNA Synthesis Kit (Clontech) and accompanying protocols were used for generating full-length cDNAs. This method relies upon template switching to an exogenous tag oligonucleotide when the reverse transcriptase reaches the 5′ end of the molecule. A retroviral reverse transcriptase was used to initiate cDNA synthesis at the 3′ poly(A) tail. Double-stranded cDNA was separated on a 1× TAE agarose gel. To enrich for larger cDNA molecules, size fractions were cut from the gel from 0.5 to 1 kb, from 1 to 2 kb, and from 2 to 3.5 kb.

The preparation of sequencing templates with a minimum of 1 μg of dsDNA input into each library procedure was performed according to the manufacturer's SMRTbell template protocol (SMRTbell Template Preparation Kit 1.0). Libraries were purified by two sequential 0.45× AMPure PB purifications (Pacific Biosciences) after exonuclease digestion of incomplete SMRTbell templates. Libraries were quantified by fluorimetry and assayed for quantity and size distribution by Bioanalyzer. Using standard protocols for C4 chemistry with the P6 enzyme, sequencing of three SMRTbell libraries for different size fractions was performed on the Pacific Biosciences *RS* II with three SMRT cells run.

### Constructs and transformation

The circRNA overexpression constructs were generated based on the vector pTCK303 backbone (Wang et al. 2004). The strategy for making the circRNA overexpression constructs is shown in Supplemental Figure 5. We first used PCR with suitable primers (repFor-F-KpnI = 5′-cggggtaccATGCATGCCAGTGAAGTGGT-3′, KpnI, and repRev-R-BamHI = 5′-cgcggatccGCGCTACATGGTGGTCAAGA-3′, BamHI) to amplify a rice intron (AK059152) with restriction sites (set in lowercase letters) for cloning. The resulting PCR products were digested by KpnI and BamHI and inversely ligated downstream from a Ubi promoter to obtain the transitional vector. Then, the circRNA locus was amplified from the cDNA using the corresponding forward/reverse primers (Circ172d-chr08.2-KpnI-F = 5′-CGGGGTACCAGTGACTTGTCGTGATTGGT-3′; Circ172d-chr08.2-PacI-R = 5′-CCTTAATTAATGTTGAAAGAAGGTCATCTTTGAG-3′) and ligated behind the rice intron using the restriction enzymes KpnI and PacI. The control construct used a nontranscribed rice sequence (AK112102, F = 5-cggggtaccGGTAAGTTACTACAAACCTTTTTG-3′, KpnI, R = 5′- cggttaattaaTGAAAATCTCGAAACAGCCGTGTC-3′, PacI) instead of circRNA. Afterward, the rice intron was amplified with another pair of primers (repFor-F-PacI = 5′-cggttaattaaATGCATGCCAGTGAAGTGGT-3′, PacI) and (repRev-R-SacI = 5′-cgcgagctcGCGCTACATGGTGGTCAAGA-3′, SacI), and ligated downstream from the circRNA sequence using PacI and SacI. The resulting circRNA overexpression constructs were transformed into rice calli (*Oryza sativa* L. ssp. *japonica*) by *Agrobacterium*-mediated transformation to generate transgenic rice plants (Hiei et al. 1994). The divergent primers used for qRT-PCR of Os08circ16564 were 5′-TACAGTGATGGGATGCTCCA-3′ and 5′-TGATATACGAATGCGGACGA-3′. The primers used for qRT-PCR of the parental gene AK064900 were 5′-AGGACAACTTGTTCCGTTGC-3′ and 5′-CACACTGGGTGCAAAAATTG-3′.

### Reads alignment and analysis

All ssRNA-seq data obtained from both leaves and panicles were preprocessed to clean the reads using the software SeqtrimNext (https://github.com/dariogf/SeqtrimNext). Sequence alignments were performed by SMALT v0.5.7 (ftp://ftp.sanger.ac.uk/pub/users/hp3) with the parameters set as “-p -i 1000 -j 45 -m 35” for mapping to the RAP-DB genome annotation data (http://rapdblegacy.dna.affrc.go.jp/download/index.html) and “-p -i 6000 -j 50 -m 35” for the rice reference genome IRGSPv5.0 (http://rgp.dna.affrc.go.jp/IRGSP/Build5/build5.html). Segemehl (Hoffmann et al. 2014), another software program, was used to detect candidate reads with the parameters set as “-S –M 1” for further comparison with our pipeline. A transcript coverage map was displayed based on the alignment results generated by the software TopHat v1.3.3 (Trapnell et al. 2009), Cufflinks v2.0.2 (Trapnell et al. 2010), and samtools v0.1.15 (http://samtools.sourceforge.net/). Referring to annotated gene models, we calculated FPKM (fragments per kilobase of exon models) for each transcript. The Pearson's χ^2^ test was then applied to assess the biological replicates. Differentially expressed genes (DEGs) were identified based on “cuffdiff” from Cufflinks v2.0.2 software. The FPKM values of the transcripts were used for comparison by computing fold changes (with absolute value ≥2) and using Fisher's exact test (*P* < 0.001). Genes were classified into different functional processes based on gene ontology (GO) term enrichment using the Web Gene Ontology Annotation Plot (WEGO) server (http://wego.genomics.org.cn/cgi-bin/wego/index.pl).

### Computational pipeline of detecting putative circularization junctions

To systematically identify rice circRNAs, a rigorous pipeline was developed. Design principles were referred to previous studies (Memczak et al. 2013; Jeck and Sharpless 2014). In the first step, partially aligned reads, that is, at least 20 bp of reads that could not be continuously mapped to the genome, were evaluated as potential chiastic reads for further analysis. For each partially aligned read, 20 bp from each end was individually extracted and independently mapped to the genome. Reads that could be perfectly, uniquely matched in a chiastic order to the same chromosome within 15 kb were selected as candidate reads. To identify putative circRNA splicing junctions, based on anchored positions, we assembled the downstream and upstream sequences of each candidate read into pseudo-genomes. Allowing for 99.8% of intron sizes to be <5 kb and 98.2% of rice gene sizes to be <10 kb (based on rice RAP-DB annotation data), the pseudo-genome size for each read was set to 10.2 kb, including 5.1 kb of downstream sequence and 5.1 kb of upstream sequence to avoid missing backsplicing sites (Fig. 1A). We then ran the genomic mapping and alignment program (GMAP) (Wu and Watanabe 2005) for each read and pseudo-genome pair to detect splicing junctions. For the detected splicing junctions, we obtained real genomic locations based on the coordinates of their corresponding pseudo-genome. To further reduce the false-discovery rate (e.g., sequencing artifacts) based on paired-end sequence data, we filtered out cases that could not be explained by a circRNA; that is, the second read in the pair mapped outside the backspliced exons. Next, we required that at least two independent reads support each breakpoint of an identified backsplicing junction and that the junctions conformed to the GU/AG intron rule.

### Simulation testing

Based on the Rice Annotation Project Database (RAP-DB) annotation data, we randomly intercepted 5′- and 3′-terminal sequences (ranging from 20 to 80 bp) from different exons and reverse-assembled them together. From the simulated reservoir, we selected candidate reads with both 20 bp parts aligned uniquely to the genome within 15 kb (anchored alignments) for further circRNA identification. We generated five individual simulated data sets to evaluate the efficiency of the circRNA detection procedure.

### Prediction of miRNA target mimics from rice circRNAs

The sequences of 713 rice miRNA precursors and mature miRNAs were downloaded from miRBase (release 21; http://www.mirbase.org/) (Griffiths-Jones et al. 2006). Our 1356 ecircRNAs were used as the target mimic prediction library. We predicted target mimics using Perl scripts with the following rules, referring to previous research (Wu et al. 2013): (i) 3-bp bulges must exist at the 5′ and ninth to twelfth positions of miRNA; (ii) except for the central bulge, no other bulge is permitted; (iii) at the 5′ end, the second to eighth positions of the miRNA must be perfectly paired to the complementary sites of the circRNA; and (iv) outside the central bulge region, at most three mismatches (G/U pair was measured as 1/2 mismatch) are permitted within the miRNA and circRNA pairs.

### Prediction of circRNAs in other plants

RNA-seq data sets were retrieved from the GEO (Gene Expression Omnibus; http://www.ncbi.nlm.nih.gov/geo/) (Barrett et al. 2009). The accession numbers of these data sets are as follows: (i) *Arabidopsis thaliana*: from ERR588038 to ERR588049; (ii) *Brachypodium distachyon*: SRR1635409, SRR1635416, and SRR1635430; (iii) *Sorghum bicolor*: from SRR1161660 to SRR1161687; (iv) *Setaria italica*: from SRR442161 to SRR442164; and (v) *Zea mays*: from SRR1656746 to SRR1656781. The genome sequences were retrieved from Gramene (TAIR 10.22 for *Arabidopsis thaliana*, version 1.0.22 for *Brachypodium distachyon*, version 1.22 for *Sorghum bicolor*, version 2.0.22 for *Setaria italica* and version 3.22 for *Zea mays*: http://www.gramene.org/archive).

## DATA DEPOSITION

All ssRNA-seq data used in this research have been submitted to the European Molecular Biology Laboratory (EMBL) Sequence Read Archive (SRA; http://www.ebi.ac.uk/sra) under accession number PRJEB8082.

## SUPPLEMENTAL MATERIAL

Supplemental material is available for this article.

## Supplementary Material

Supplemental Material
