# Supplementary material for: Transcriptome-wide investigation of circular RNAs in rice
Source: RNA. 2015 Dec;21(12):2076–87. doi: 10.1261/rna.052282.115 (PMC4647462; doi:10.1261/rna.052282.115)
Supplement: Supplemental Material [file supp_21_12_2076__index.html]

Transcriptome-wide investigation of circular RNAs in rice — Transcriptome-wide investigation of circular RNAs in rice — Supplemental Material 

# Transcriptome-wide investigation of circular RNAs in rice

## Supplemental Material

**Files in this Data Supplement:**

- Supp Figure S5.pdf
- Supp Figure S4.tif
- Supp Legends.docx
- Supp Figure S1.tif
- Supp Figure S3.tif
- Supp Table S2.xlsx
- Supp Table S6.xlsx
- Supp Figure S2.tif
- Supp Table S3.xlsx
- Supp Tables S1, S4, S5.docx
