# Supplementary material for: Transcriptome-wide investigation of circular RNAs in rice
Source: RNA. 2015 Dec;21(12):2076–87. doi: 10.1261/rna.052282.115 (PMC4647462; doi:10.1261/rna.052282.115)
Supplement: Supplemental Material [file supp_052282.115_FigS5.pdf]

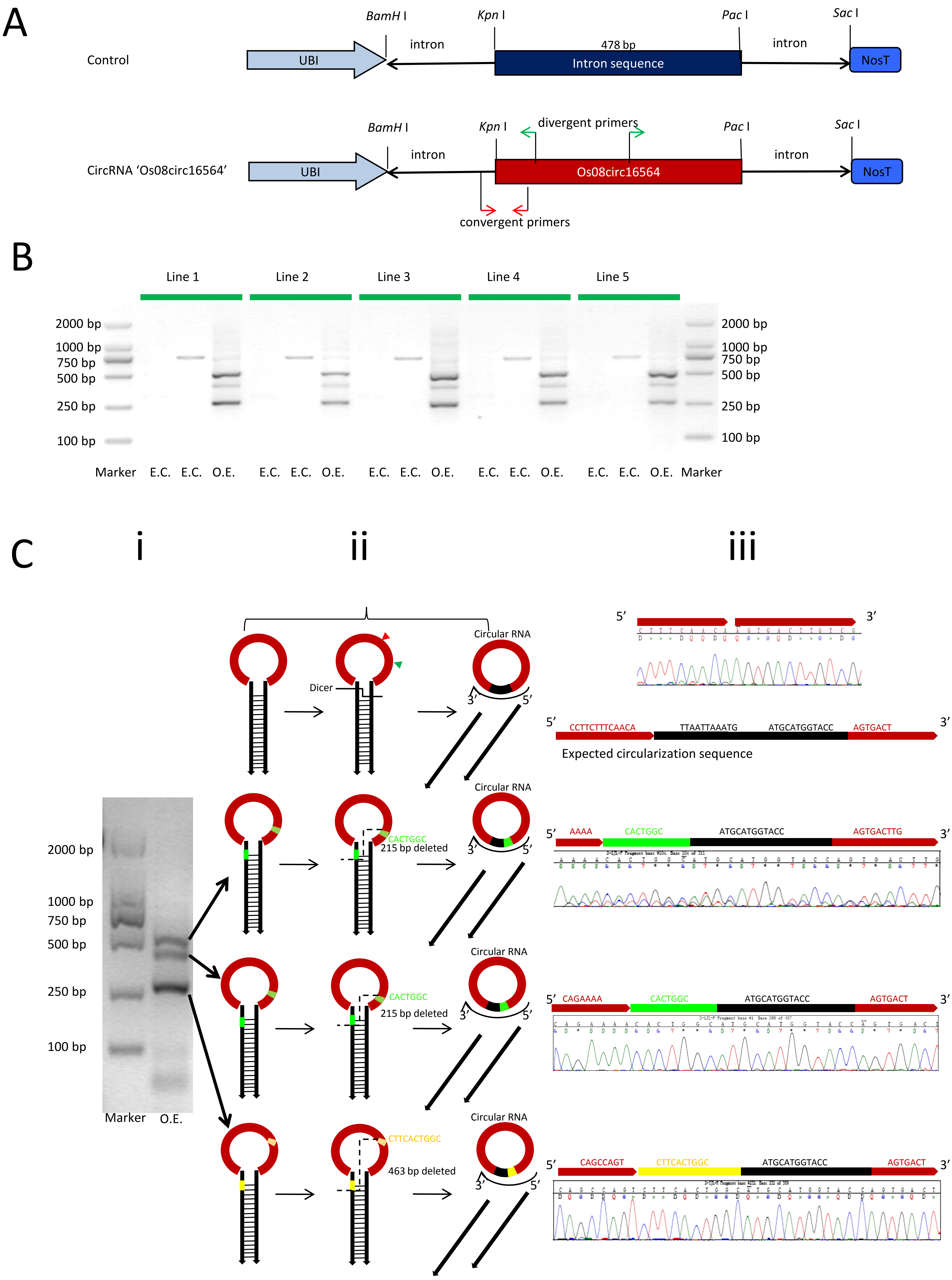

Supplemental Figure 5. Detailed information of overexpression circRNA constructs. (A) Schematic diagram of overexpression circular RNA construct. A 478-bp rice intron (dark blue box) of control construct or 1096-bp Os08circ16564 exons fragment (red box) was used to generate the overexpression construct in the pTCK303 vector with *UBI* promoter, *Nos* terminator (*Nos*T) and restriction enzyme sites of *Kpn* I, *Bam*H I, *Pac* I and *Sac* I. DNA fragments (black arrows) from a rice gene intron that was not expressed was ligated into both upstream and downstream flanking Oscirc16564 exons in an orientation-opposite pattern. *Ubi*, maize (*Zea mays*) ubiquitin; *Nos*T, nopaline synthase terminator. Convergent primers (red arrows) and divergent primers (green arrows) used in the quantitative RT-PCR were indicated. (B) RT-PCR products by divergent primers. Each line was indicated by green bar. As for every line, the first and the second lanes showed the results of control plant. The second lane was the nest PCR products that used the first PCR products as templates. The third lane showed the divergent PCR products of overexpression line. E.C.; empty vector transgenic control. O.E.; overexpression. (C) Validation of transgene circRNA 'Os08circ16564' expression. (i) Os08circ16564 expression in transgenic plant (right lane). DNA markers are indicated (left lane). (ii) Schema designed for an overexpression vector that produces circRNA. Up row: expected circRNA structure. Following down rows: circRNA structures experimentally detected. Red and black boxes represented circRNA and intron sequences, respectively. Light green and yellow represented dissecting sites from hairpins that were homologous with that (dark green and orange) of circRNAs. The dotted lines indicated the real dissection sites. Vertical triangles indicated the predicted miRNA osa-miR810b.2 (red) and osa-miR172d-5p (green) binding sites. (iii) 3730 Sanger sequencing results of three circular sequences of PCR products. Up row: expected circular sequences. Following down rows: sequencing results of three circular products. Red, light green, yellow and black boxes corresponded to the regions showed in (ii). The sequencing trace files of the junction reads of circRNA and hairpins were shown.
