# Supplementary figures and images for: Transcriptome-wide investigation of circular RNAs in rice
Source: RNA. 2015 Dec;21(12):2076–87. doi: 10.1261/rna.052282.115 (PMC4647462; doi:10.1261/rna.052282.115)

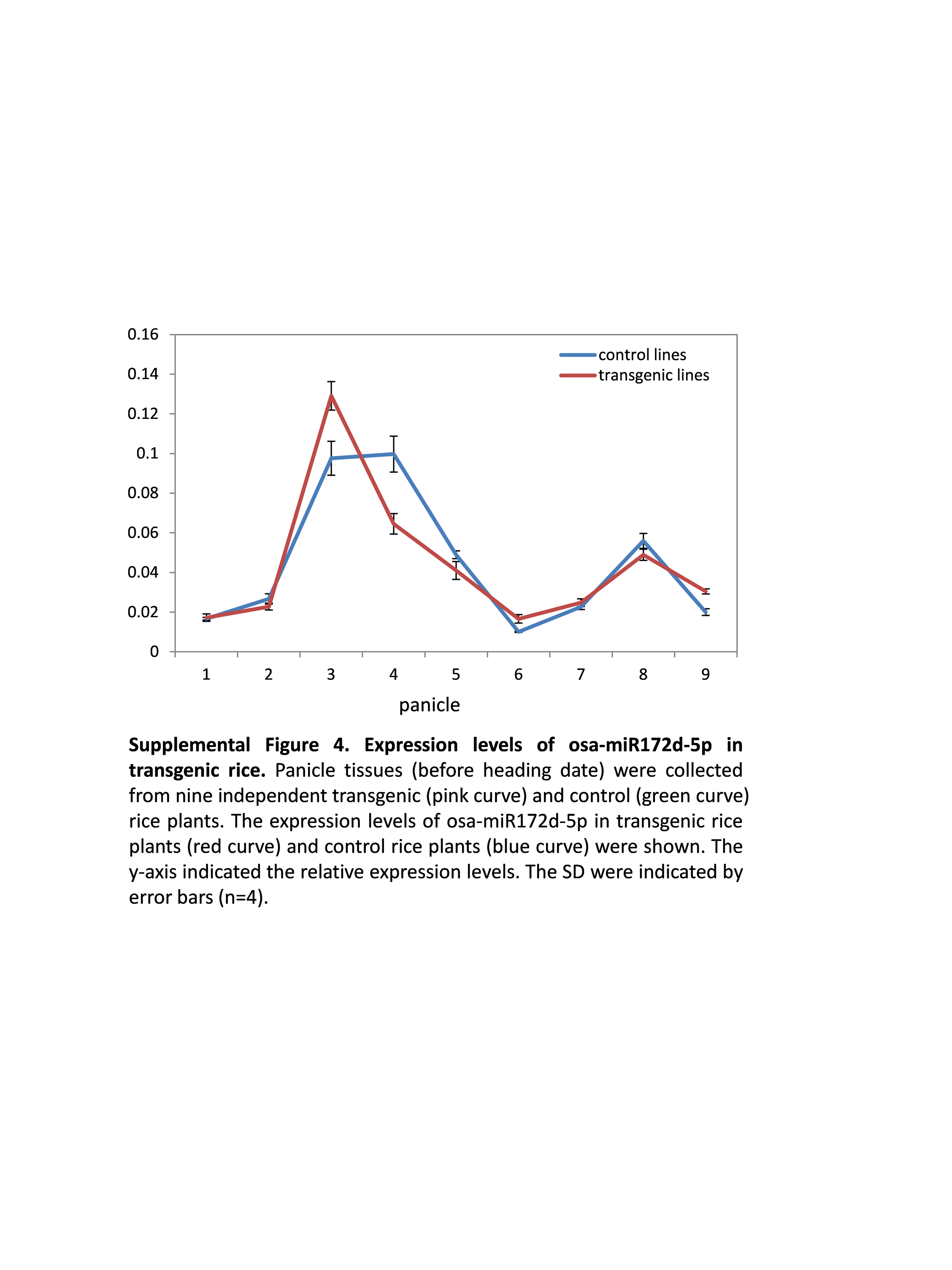

Supplement: Supplemental Material [file supp_052282.115_FigS4.tif]

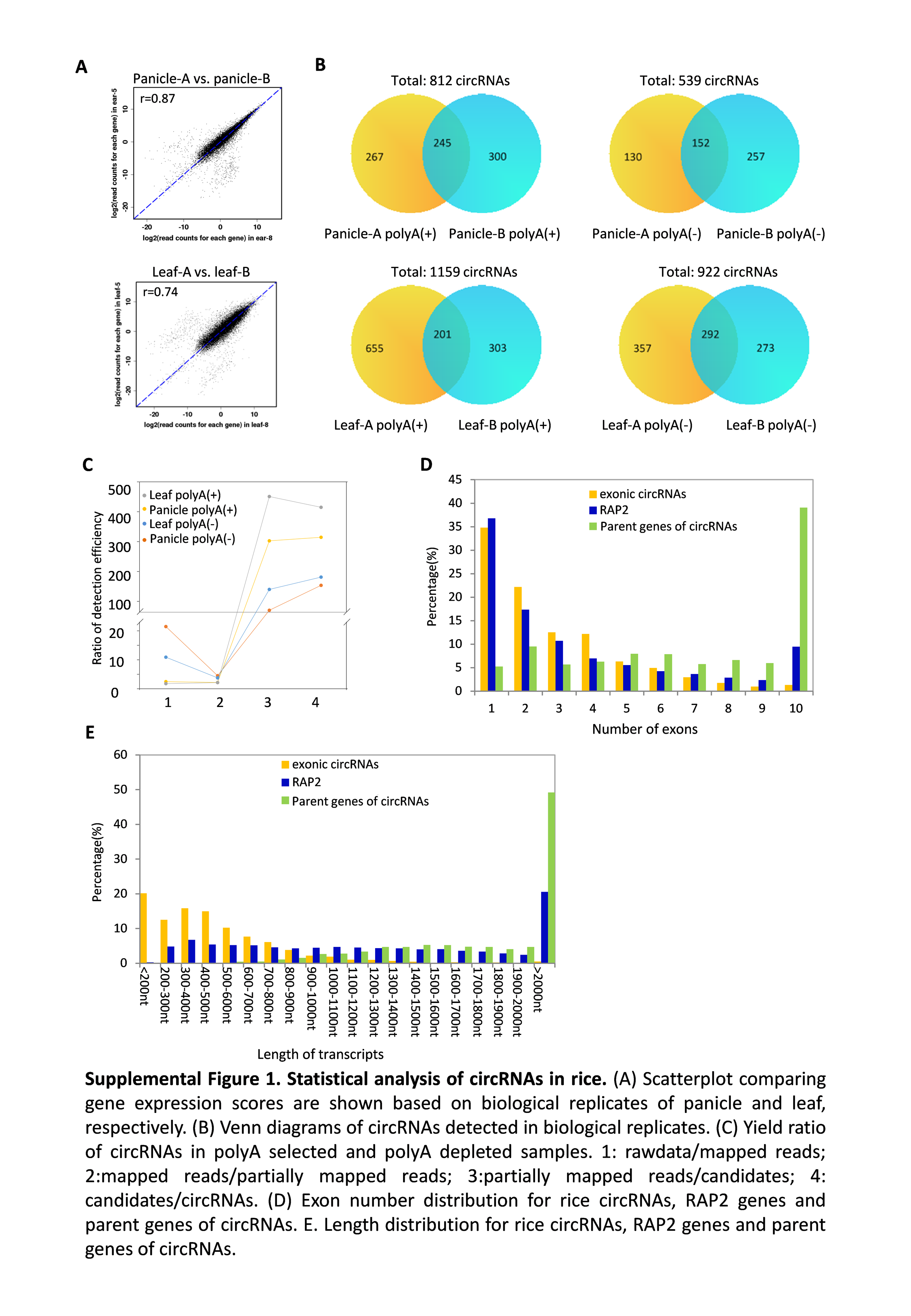

Supplement: Supplemental Material [file supp_052282.115_FigS1.tif]

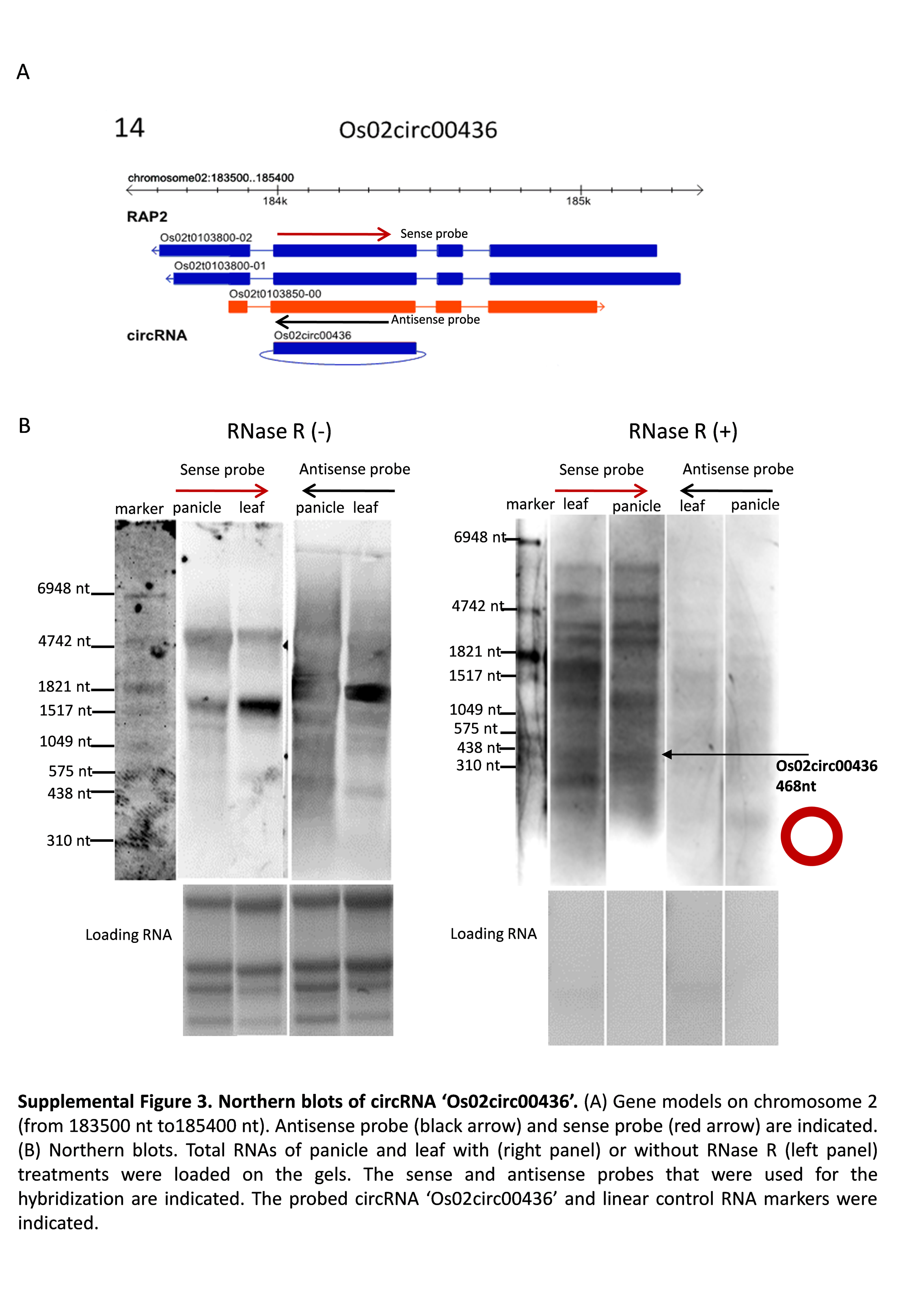

Supplement: Supplemental Material [file supp_052282.115_FigS3.tif]
