# Supplementary material for: Transcriptome-wide investigation of circular RNAs in rice
Source: RNA. 2015 Dec;21(12):2076–87. doi: 10.1261/rna.052282.115 (PMC4647462; doi:10.1261/rna.052282.115)
Supplement: Supplemental Material [file supp_052282.115_SuppLegends.docx]

**Supplementary Figure 1.** Statistical analysis of circRNAs in rice. (A) Scatterplots comparing gene expression scores are shown based on biological replicates of panicle and leaf. (B) Venn diagrams of circRNAs detected in biological replicates. (C) Yield of circRNAs in polyA-selected and polyA-depleted samples. 1: raw data/mapped reads; 2: mapped reads/partially mapped reads; 3: partially mapped reads/candidates; 4: candidates/circRNAs. (D) Exon number distribution for rice circRNAs, RAP2 genes and parental genes of circRNAs. E. Length distribution for rice circRNAs, RAP2 genes and parental genes of circRNAs.

**Supplementary Figure 2.** Experimental analysis of circRNAs in rice. We experimentally tested our rice circRNA predictions. A total of 30 out of 35 circRNAs were successfully validated. Each green box contains one circRNA’s analyzed information. Consider the first circRNA as an example (Os03circ07398): at the top left corner, the number ‘1’ is its Unique Sequence Number in this study. The upper diagram represents the gene structure of the circRNA and its parental gene. A red bar denotes an exon, and its transcriptional orientation is consistent with the reference genome, while a blue bar represents an exon with its transcriptional orientation opposite from the reference. A red or blue line denotes an intron. In the middle, each backsplice junction is displayed by a trace file. Light blue and pink indicate the joint sequence. The lower diagram of each green box shows the PCR amplification results. Divergent primers (black back-to-back triangle pairs) and convergent primers (black opposing triangle pairs) were designed to amplify each circRNA in the total RNA sample and the genomic DNA of the leaf and panicle.

**Supplementary Figure 3.** Northern blots of circRNA ‘Os02circ00436’. (A) Gene models on chromosome 2 (from 183500 nt to185400 nt). Antisense probe (black arrow) and sense probe (red arrow) are indicated. (B) Northern blots. Total RNAs of panicle and leaf with (right panel) or without RNase R (left panel) treatments were loaded on the gels. The sense and antisense probes that were used for the hybridization are indicated. The probed circRNA ‘Os02circ00436’ and linear control RNA markers were indicated.

**Supplementary Figure 4.** Expression levels of osa-miR172d-5p in transgenic rice. Panicle tissues (before heading date) were collected from nine independent transgenic (pink curve) and control (green curve) rice plants. The expression levels of osa-miR172d-5p in transgenic rice plants (pink curve) and control rice plants (green curve) were shown. The y-axis indicated the relative expression levels. The SD were indicated by error bars (n=4).

**Supplemental Figure 5.** Detailed information of overexpression circRNA constructs. (A) Schematic diagram of overexpression circular RNA construct. A 478-bp rice intron (dark blue box) of control construct or 1096-bp Os08circ16564 exons fragment (red box) was used to generate the overexpression construct in the pTCK303 vector with *UBI* promoter, *Nos* terminator (*Nos*T) and restriction enzyme sites of *Kpn* I, *Bam*H I, *Pac* I and *Sac* I. DNA fragments (black arrows) from a rice gene intron that was not expressed was ligated into both upstream and downstream flanking Oscirc16564 exons in an orientation-opposite pattern. *Ubi*, maize (*Zea mays*) ubiquitin; *Nos*T, nopaline synthase terminator. Convergent primers (red arrows) and divergent primers (green arrows) used in the quantitative RT-PCR were indicated. (B) RT-PCR products by divergent primers. Each line was indicated by green bar. As for every line, the first and the second lanes showed the results of control plant. The second lane was the nest PCR products that used the first PCR products as templates. The third lane showed the divergent PCR products of overexpression line. E.C.; empty vector transgenic control. O.E.; overexpression. (C) Validation of transgene circRNA ‘Os08circ16564’ expression. (i) Os08circ16564 expression in transgenic plant (right lane). DNA markers are indicated (left lane). (ii) Schema designed for an overexpression vector that produces circRNA. Up row: expected circRNA structure. Following down rows: circRNA structures experimentally detected. Red and black boxes represented circRNA and intron sequences, respectively. Light green and yellow represented dissecting sites from hairpins that were homologous with that (dark green and orange) of circRNAs. The dotted lines indicated the real dissection sites. Vertical triangles indicated the predicted miRNA osa-miR810b.2 (red) and osa-miR172d-5p (green) binding sites. (iii) 3730 Sanger sequencing results of three circular sequences of PCR products. Up row: expected circular sequences. Following down rows: sequencing results of three circular products. Red, light green, yellow and black boxes corresponded to the regions showed in (ii). The sequencing trace files of the junction reads of circRNA and hairpins were shown.
