# Supplementary material for: Transcriptome-wide investigation of circular RNAs in rice
Source: RNA. 2015 Dec;21(12):2076–87. doi: 10.1261/rna.052282.115 (PMC4647462; doi:10.1261/rna.052282.115)
Supplement: Supplemental Material [file supp_052282.115_TableS1_4_5.docx]

**Supplementary Table 1. Summary of ssRNA-seq experiments and data analysis.** The data shows that rice mature leaf and panicle were individually sequenced by extracting mRNA samples with polyA selected and polyA depleted treatments. Biological and technical replicates were carried out for each sample. Here, partially mapped reads means those reads which were uniquely but partially (20bp<continuous mapped length<100bp) mapped to the genome. Candidate reads means those reads which both terminal parts (20bp) could be perfectly anchored to the genome in a permuted, chiastic order.

| Sample | Replicates | Raw paired-end reads | Mapped genome | Partially mapped reads | Candidate reads | circRNAs |
| --- | --- | --- | --- | --- | --- | --- |
| Mature leaf (polyA+) | Leaf-A Replicate1 | 123,120,011 | 47,309,104 | 59,888,518 | 214,083 | 1159 |
|  | Leaf-A Replicate2 | 124,059,378 | 48,194,151 |  |  |  |
|  | Leaf-A Replicate3 | 111,638,813 | 43,145,584 |  |  |  |
|  | Leaf-B Replicate1 | 165,670,126 | 61,435,726 | 50,420,898 | 150,388 |  |
|  | Leaf-B Replicate2 | 159,050,738 | 58,980,649 |  |  |  |
| Panicle (polyA+) | Panicle-A Replicate1 | 147,095,970 | 74,100,982 | 62,241,895 | 177,963 | 812 |
|  | Panicle-A Replicate2 | 145,218,963 | 72,533,373 |  |  |  |
|  | Panicle-B Replicate1 | 98,158,116 | 52,089,841 | 89,625,769 | 158,873 |  |
|  | Panicle-B Replicate2 | 93,400,876 | 49,037,615 |  |  |  |
|  | Panicle-B Replicate3 | 199,128,416 | 102,794,842 |  |  |  |
| Mature leaf (polyA-) | Leaf-A Replicate1 | 207,396,249 | 9,359,566 | 5,370,559 | 74,345 | 922 |
|  | Leaf-A Replicate2 | 209,609,014 | 9,453,313 |  |  |  |
|  | Leaf-A Replicate3 | 176,602,934 | 8,265,461 |  |  |  |
|  | Leaf-B Replicate1 | 207,413,788 | 9,725,825 | 4593746 | 67,406 |  |
|  | Leaf-B Replicate2 | 211,764,917 | 9,972,498 |  |  |  |
| Panicle (polyA-) | Panicle-A Replicate1 | 120,155,545 | 12,203,493 | 6,261,209 | 42,796 | 539 |
|  | Panicle-A Replicate2 | 127,757,016 | 12,342,724 |  |  |  |
|  | Panicle-B Replicate1 | 175,130,662 | 14,362,962 | 7,421,366 | 54,903 |  |
|  | Panicle-B Replicate2 | 175,016,454 | 15,058,278 |  |  |  |
| Total | | 2,977,387,986 | 710,365,987 | 285,823,960 | 940,757 | 2354 |

**Supplementary Table 4. circRNAs identification in rice and other plants.**

| Species | Tissue | Reads (No.) | Read length (bp) | Genome Coverage | Candidate reads (No.) | circRNAs | Unique circRNAs (depth>=2) |
| --- | --- | --- | --- | --- | --- | --- | --- |
| Oryza sativa | Panicle-A polyA(+) | 584,629,866 | 100 | 152.7 | 177963 | 512 | 2,354 |
|  | Panicle-B polyA(+) | 781,374,816 |  | 204.1 | 158873 | 545 |  |
|  | Leaf-A-polyA(+) | 717,636,404 |  | 187.5 | 214083 | 856 |  |
|  | Leaf-B-polyA(+) | 649,441,728 |  | 169.7 | 150388 | 504 |  |
|  | Panicle-A polyA(-) | 495,825,122 |  | 129.5 | 42796 | 282 |  |
|  | Panicle-B polyA(-) | 700,294,232 |  | 182.9 | 54903 | 409 |  |
|  | Leaf-A-polyA(-) | 1,187,216,394 |  | 310.1 | 74345 | 649 |  |
|  | Leaf-B-polyA(-) | 838,357,410 |  | 219.0 | 67406 | 565 |  |
| Brachypodium distachyon | Shoots (Mock-inoculated) | 40,504,332 | 100 | 14.8 | 9999 | 18 | 26 |
|  | Shoots (PMV-infected) | 41,140,892 |  | 15.1 | 8592 | 21 |  |
|  | Shoots (PMV+SPMV-infected) | 37,977,424 |  | 13.9 | 10,034 | 22 |  |
| Sorghum bicolor | root (CK60) | 33,250,650 | 76 | 3.4 | 11,373 | 34 | 75 |
|  | root (TX623) | 29,691,142 |  | 3.1 | 9,857 | 25 |  |
|  | root (SCS) | 29,851,268 |  | 3.0 | 8,922 | 30 |  |
|  | root (Ch17) | 30,783,227 |  | 3.1 | 9,847 | 32 |  |
|  | root (KS78) | 33,075,152 |  | 3.4 | 10,338 | 20 |  |
|  | root (High Bulk) | 29,318,230 |  | 3.0 | 8,884 | 22 |  |
|  | root (Low Bulk) | 31,628,173 |  | 3.2 | 10,667 | 20 |  |
| Setaria italica | root | 38,487,562 | 90 | 8.5 | 3,669 | 98 | 113 |
|  | leaf | 38,399,466 |  | 8.5 | 3,511 | 88 |  |
|  | stem | 38,519,470 |  | 8.5 | 5,221 | 164 |  |
|  | spica (tassel) | 39,251,372 |  | 8.7 | 5,079 | 128 |  |
| Zea Mays | B73 leaf control 24hr | 82,928,588 | 100 | 4.010349883 | 28,211 | 247 | 496 |
|  | B97 leaf control 24hr | 95,318,678 |  | 4.609523186 | 17,426 | 178 |  |
|  | Mo18w leaf control 24hr | 100,046,858 |  | 4.838173601 | 38,324 | 217 |  |
|  | M162w leaf control 24hr | 112,673,010 |  | 5.448762645 | 50,431 | 300 |  |
|  | B73 leaf 24hr | 104,637,761 |  | 5.1 | 35,299 | 295 |  |
|  | B97 leaf 24hr | 126,117,546 |  | 6.1 | 38,968 | 225 |  |
|  | Mo18w leaf 24hr | 113,748,899 |  | 5.5 | 36,467 | 203 |  |
|  | M162w leaf 24hr | 103,375,377 |  | 4.9 | 21,783 | 166 |  |
|  | B73 leaf 72hr | 101,628,545 |  | 4.9 | 46,180 | 313 |  |
|  | B97 leaf 72hr | 104,750,135 |  | 5.1 | 25,962 | 206 |  |
|  | Mo18w leaf 72hr | 97,788,231 |  | 4.7 | 7,525 | 137 |  |
|  | M162w leaf 72hr | 94,698,042 |  | 4.6 | 14,903 | 164 |  |
| Arabidopsis thaliana | seed | 151,938,660 | 150 | 190.4 | 20,624 | 278 | 165 |
|  | siluge without seeds, grown at 16 degree Celcius | 113,386,188 | 100 | 94.7 | 24,535 | 152 |  |
|  | siluge without seeds,grown at 22 degree Celsius | 125,308,176 |  | 104.7 | 20,925 | 132 |  |

**Supplementary Table 5. Primer list of 30 validated circRNAs.**

| No. | Name of circRNA | Classification | Backsplice | Parent Gene | Divergent primers | Convergent primers |
| --- | --- | --- | --- | --- | --- | --- |
| 1 | Os03circ07398 | exonic | both known junctions | Os03t0220700-02 | P1: TCGCCATACTCCTATACACACG  P2: ACATCCGGTTGAGCTAGAGAAC | P1: CAAACCGAGGAAGCAATAAACT  P2: CCAGCAAAACGTGTGTATAGGA |
| 2 | Os05circ03807 | exonic | either known junction | Os05t0161200-01 | P1: AAAGACGATCGCTACAGGAAGA  P2: TAGATTACCAGCTCCAACAGCA | P1: AAATGGTTACCAGAGCTTGCAT  P2: GTTTGCGGTAGACCTGAATGAG |
| 3 | Os09circ02967 | exonic | both known junctions | Os09t0343200-01 | P1: TAACATGAGATGTTGGGACCTG  P2: CAGACGGGAATCTTTCGTACTC | P1: TTTTGTCTATTTGGGCCAGAGT  P2:TCCCAACATCTCATGTTAGTGC |
| 4 | Os01circ20329 | exonic | both known junctions | Os01t0680700-01 | P1: GCTTGGAATCGAGCTGTGAC  P2: AACCTGAGGTCCGCTTCGT | P1:GCTTGGAATCGAGCTGTGA  P2:AGGAAGCGGTAGCGTGACA |
| 5 | Os09circ11453 | exonic | both known junctions | Os09t0568000-02 | P1: GCTGGATCAAGTGAAATGATGA  P2: AGCATCATGAGAGCAGCAGATA | P1:GGCCTACAGAATCCAGCATATC  P2: ACTGAAGATTGAAGCAGCAACA |
| 6 | Os02circ30975 | exonic | either known junction | Os02t0828200-01 | P1: AGGTGCTTCTGAACATGGAGAT  P2: TTGCTATGCTTTCGTTGAGCTA | P1:TCGCGAGGAAGAGATACACATA  P2: TGAATGGGTTCACTCACTTGAC |
| 7 | Os05circ03983 | exonic | both known junctions | Os05t0164100-01 | P1: GTGTATGAGCCAGTGCAGGA  P2: TACAGTTCCCTTCAGCCATTTT | P1:TTTGATGCGACAAACAGCACA  P2:CTGGCTCATACACCTTTTCATAGT |
| 8 | Os06circ09294 | exonic | either known junction | Os06t0340600-01 | P1: TCATGCAGGGAACATTTTAGTG  P2: GAGAACCCGATACGTTCTTCAG | P1: GTGTTTAAGCCCAGAGATGAGG  P2:CACTAAAATGTTCCCTGCATGA |
| 9 | Os02circ19718 | exonic | both novel junctions | Os02t0634500-01 | P1: AGGTGAAACCTTTTCGTGTCC  P2: GGTTCTCAATTACCTCGTCGAT | P1:TCATCGACGAGGTAATTGAGAA  P2:ATCGGCAACCTGAATAACAAGT |
| 10 | Os04circ13410 | exonic | both novel junctions | Os04t0610800-01 | P1: TCTGTAGCCTTACGATCTCCAA  P2: GAACGAAATCAAGCTGTTCAGA | P1:GTTTGTTAGCTTTCCTCGCACT  P2:GCTCACCGGCTATATTTCATTC |
| 11 | Os03circ20866 | Across 2 Gene | either known junction | Os03t0641700-01 and Os03t0641700-02 | P1: ATCGCCATAAAGATCGGAGTAA  P2: AAGGCAAATCTATTGGTGCTGT | P1:CTTTGACATGGAAACACTTGGA  P2:GATGCGCACTCTTATCCTTCTT |
| 12 | Os06circ02797 | exonic | both novel junctions | Os06t0137600-01 | P1: ATTAAAGGAGGAAAGGGAGCAA  P2: ACTCATCCTCGATGAACCGTAT | P1:AGTACGTCAGGAGCCAAATAGG  P2:TGATAGATTTGCACCTTCTCCA |
| 13 | Os11circ09617 | exonic | both novel junctions | Os11t0685600-00 | P1: TGGTGTGTAGATTGGATCTTCG  P2: CTGCTGCAACTATTAGCGTCTG | P1: CAGACGCTAATAGTTGCAGCAG  P2: CGAAGATCCAATCTACACACCA |
| 14 | Os02circ00436 | exonic | both known junctions | Os02t0103800-01 | P1: TTGAACTTGTAGTCGTCGTGCT  P2: AGTTCGAGCGGATGAAGGAG | P1:CCTTCAGGTCCTTCCTGTGGA  P2:ATGTCGTCGATGCCTTTCTCC |
| 15 | Os01circ31126 | intergenic | both novel junctions | Intergenic region | P1: GATCTCCTGGAACGTCTCCTT  P2: ATCGAACCTGCTGTGTGTACC | P1:TAAGCCACCACATGACCACC  P2:GGAGACGTTCCAGGAGATCG |
| 16 | Os07circ08968 | exonic | both known junctions | Os07t0507700-01 | P1: CCTTGCGATGATGCTTCTACTT  P2: AATCCATCCACTTTTGTGATCC | P1:CCTTATGAGTTTGCAGCAGATG  P2:TTGTCTGGAAGAAGCAAGAGGT |
| 17 | Os03circ25430 | exonic | both known junctions | Os03t0732100-01 | P1: CCACCACATGGGGATCAT  P2: AGTCGAAGAAGTTCACCACCAT | P1:ACAACCACTACTGCGACCAG  P2:GATCCCCATGTGGTGGAAGG |
| 18 | Os05circ14065 | exonic | both known junctions | Os05t0492500-01 | P1: TATGAGATGGATGACCTGCTTG  P2: AAGTGACGAGTCAAGTCTGCAA | P1:GGATCGGAGGACTTCAGCAG  P2:TGTGCTAATTTAACTGCTGTTCCG |
| 19 | Os08circ07854 | exonic | either known junction | Os08t0377400-01 | P1: AACACGGTAAATCACCAACCTC  P2: TGGAGCCACTAATTCTGGAGAT | P1:TCAACACCGAGCAGGTGAAC  P2:CTGGCACTGAGCGTCATCTC |
| 20 | Os02circ25329 | intronic | both novel junctions | Os02t0734600-01 | P1: GGGATCGATGGTCTCATGG  P2: GCATTCACCTCTTCCTTTGTGT | P1:CTGCTTCGTTTCAGAGAGAGG  P2:CACCAGCACCCTACTTACGAT |
| 21 | Os07circ21701 | exonic | both known junctions | Os07t0695100-01 | P1: AGTGGCAGTGGAAGTGAAAGT  P2: TGTGGTTCATGATCCTACTCAA | P1: TTGTTATGCCTGGTGTATCTGG  P2: CACTGCCATCTCTTGCATTAAG |
| 22 | Os05circ09155 | exonic | both novel junctions | Os05t0381500-01 | P1: GTAACACGGGCTACGAGAAGTA  P2: AGTGCACTCTGCCTTTGTCTTT | P1: AATATACGCCTTGGTGGAGAGA  P2: CGTCTAGGGCAGCGTAGTAGTT |
| 23 | Os03circ00204 | intergenic | both novel junctions | Intergenic region | P1: CAAACAGGCTCTCAGTGACAAG  P2: TGATGCTACTGTCTACGGTCCA | P1:CTTTTATGGGCATTCAGAGCTT  P2:TCTGATTTCAGCTTCCTTGTCA |
| 24 | Os03circ04984 | exonic | both known junctions | Os03t0180300-01 | P1: CAGTGCAAAACCTTACTGATGC  P2: GTCCCAGAACTTTGAAATCAGG | P1:ACTCTGAGCACACGATCAAAGA  P2:TCCGAAGTATGCATCAGTAAGGT |
| 25 | Os01circ14411 | intergenic | both novel junctions | Intergenic region | P1: CATTGCTGGCTTTTAAGACCTC  P2: ATTCTACGTCCTTGCCTCACAT | P1:ATGTGAGGCAAGGACGTAGAAT  P2:CTCTTCATTGAGCTTTCACACG |
| 26 | Os01circ08024 | exonic | both known junctions | Os01t0259200-01 | P1: GCCAAGAACCACTAGATTGGAC  P2: TGCTTTATACACACTGCCGAAT | P1: GGGTCGATAGATATGGTTGGAA  P2: CATTATACCCTTCGCCAAGAAG |
| 27 | Os02circ11223 | exonic | both known junctions | Os02t0302200-01 | P1: ATAATGGTGATCAGCGACGAG  P2: GTTGTTGGGGTTGACGATG | P1:GTGGCGGCGCACCTGTCG  P2:CCTCGTCGCTGATCACCATT |
| 28 | Os02circ07060 | exonic | both known junctions | Os02t0194700-01 | P1: CTTCACGTCCGAGAACTGCT  P2: CACCTCCACCAACATCGTC | P1:AGCAGTTCTCGGACGTGAAG  P2:GACGATGTTGGTGGAGGTG |
| 29 | Os07circ20289 | exonic | both known junctions | Os07t0672500-02 | P1: GGAAAGATCAAGCACATCAACA  P2: TGATCCAGGAAATGACTCTGTC | P1:GAGGCTGACAGAGTCATTTCCT  P2:AAGGGCATTACTGATAGCTTGG |
| 30 | Os05circ02465 | intergenic | both novel junctions | Intergenic region | P1: ctccattccattttgcagtg  P2: tcaatgtgatttggatgcttg | P1: CTTTTCTTCCGCAGCTGTCTAT  P2: AGTTGAAAGCAAAAGGCCACT |
| 31 | 14sense-F | | | | GCGTAATACGACTCACTATAGGGAGAGATCATGATGTCGTCGATGC | |
| 32 | 14sense-R | | | | cttcaggtccttcctgtgga | |
| 33 | 14antisense-F | | | | GCGTAATACGACTCACTATAGGGAGActtcaggtccttcctgtgga | |
| 34 | 14antisense-R | | | | GATCATGATGTCGTCGATGC | |
| 35 | osa-miR172d-5p-F | | | | AGCTGGGCAGCACCATCA | |
| 36 | osa-miR172d-5p-R | | | | GCAGGGTCCGAGGTATTC | |
| 37 | osa-miR172d-stem-loop RT primer | | | | GTCGTATCCAGTGCAGGGTCCGAGGTATTCGCACTGGATACGACGTGAATCT | |
| 38 | UBQ5-QRT-F | | | | ACCACTTCGACCGCCACTACT | |
| 39 | UBQ5-QRT-R | | | | ACGCCTAAGCCTGCTGGTT | |
| 40 | Os08circ16564_conF | | | | CACTGGCATGCATGGTACCAGTG | |
| 41 | Os08circ16564_conR | | | | CCTGAGTTTAGAAACATCGGCTA | |
| 42 | actin-QRT-F | | | | GCCGTCCTCTCTCTGTATGC | |
| 43 | actin-QRT-R | | | | GCCGTTGTGGTGAATGAGTA | |
| 44 | actin-RT-F | | | | CCTTCAACACCCCTGCTATG | |
| 45 | actin-RT-R | | | | CAATGCCAGGGAACATAGTG | |
